# Embryonic development grand challenge: crosslinking advances

**DOI:** 10.3389/fcell.2024.1467261

**Published:** 2024-09-19

**Authors:** Beate Brand-Saberi

**Affiliations:** Department of Anatomy and Molecular Embryology, Institute of Anatomy Universitaetsstrasse, Ruhr University Bochum, Bochum, Germany

**Keywords:** embryoid, oxidative stress, malformation, higher education, organoid, 3Rs (reduce replace refine), transcriptomics, cell atlas studies

## Abstract

Research on embryonic development is entering into a new era. As a traditionally descriptive discipline within anatomy, embryologists have formed international consortia and digitized important histological collections for preservation and open access. Embryonic development has recently received a wider attention in context with temporo-spatial transcriptomics at single cell level. These can be expected to fuel the realization of the transdisciplinary significance of efforts to decipher embryonic development. Addressing its complexities encompasses a wealth of challenges that intersect across the domains of science, society, and politics underlining its outstanding importance as well as its inherently interdisciplinary nature. The challenges of this field are by no means confined to understanding the intricate biological mechanisms but also have humanitarian implications. To fully appreciate the mechanisms underlying human development, principles of embryogenesis have predominantly been analyzed employing animal models which allow us to broaden our view on developmental processes. As a result of recent pioneering work and technical progress centered around stem cell-based 3D approaches, we are entering into a historical new phase of learning about mammalian embryonic development. In vertebrates, a growing concern now focuses the reduction of animal experimentation. This perspective article outlines the major challenges in this amazing field that offer an enormous potential for basic biomedical sciences as well as related translational approaches if they are tackled in a multidisciplinary discourse.

## Introduction

**FIGURE 1:**
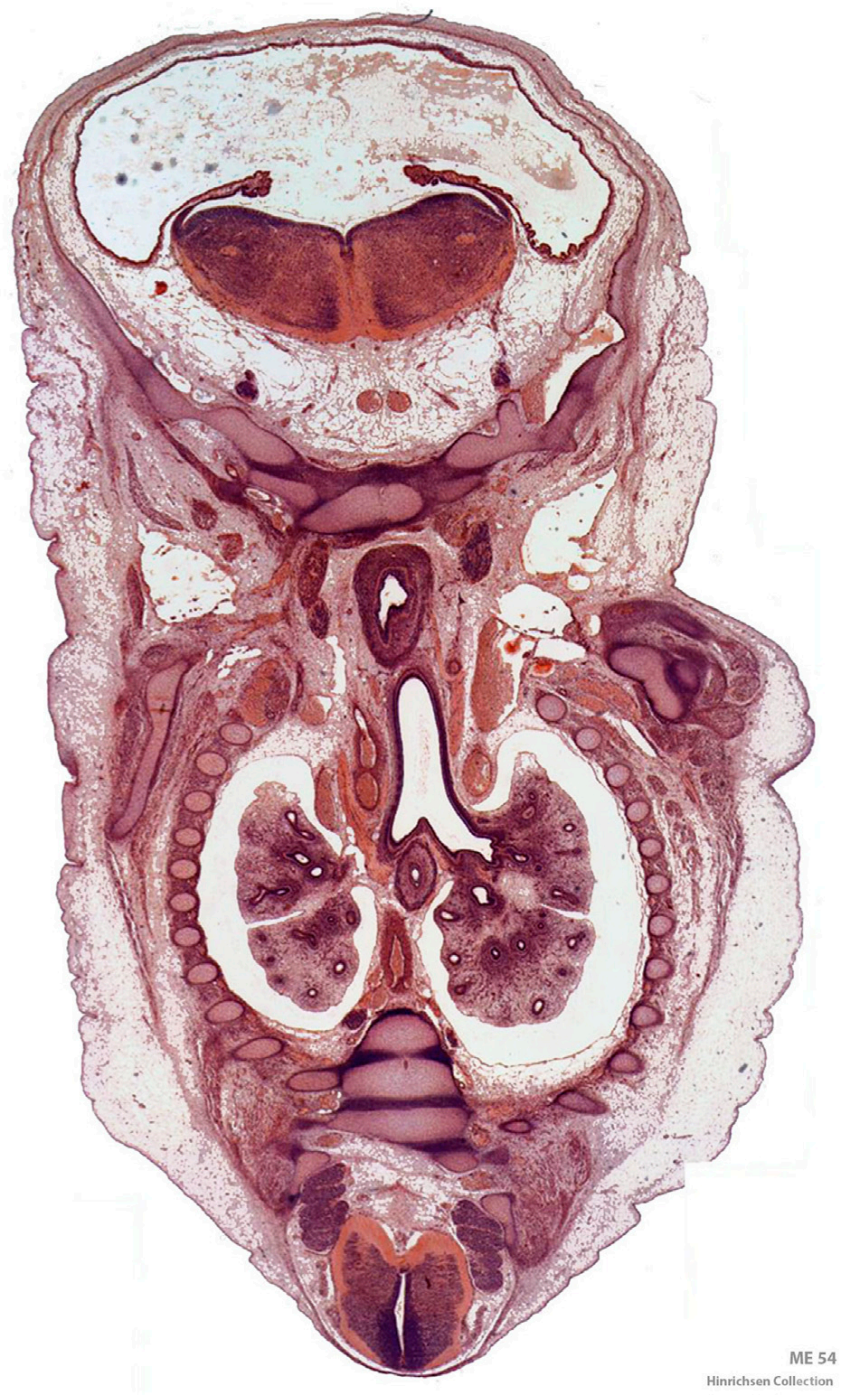
Digitized frontal section of a Carnegie stage 21 (22.5 mm) human embryo from Professor Klaus Hinrichsen’s histological collection of human embryos at Ruhr University Bochum, Germany. The thoracic cavity with pleural sacs and the developing lungs is in the center of the section. The esophagus is located ventral to the tracheal bifurcation. The section passes through the skull base cranially with myelencephalon and fourth ventricle and the caudal part of the spinal cord at the opposite end (Hill, 2024).

Embryonic development has moved forward as a discipline that is entering into a new complex and amazing phase. As a traditionally descriptive discipline within anatomy, embryologists have assembled into international consortia (Brand-Saberi et al., 2012) and digitized important histological collections of high quality (Hill, 2019; Maricic et al., 2019; Figure 1). Embryology has recently received a wider attention in context with temporo-spatial transcriptomics at single cell level such as the human cell atlas project (Zhang et al., 2023; https://www.humancellatlas.org) that will help tremendously in the realization of the transdisciplinary significance of efforts to decipher embryonic development. Addressing its complexities encompasses a wealth of challenges that intersect across the domains of science, society, and politics underlining its outstanding importance as well as its inherently interdisciplinary nature. The challenges of this field are by no means confined to understanding the intricate biological mechanisms but extend to ethical, legal, social and philosophical implications. To fully appreciate the mechanisms underlying human development, principles of embryogenesis have been mainly analyzed employing animal models which allow us to widen our view on developmental processes. As a result of recent pioneering work and technical progress centered around stem cell-based 3D approaches, we are entering into a historical new phase of learning about mammalian embryonic development (Dupont, 2024). Simultaneously, ethical concerns strive to reduce the extent of animal experimentation, particularly of vertebrates.

This perspective article outlines the major challenges in this amazing field that offer an enormous potential for basic biomedical sciences as well as related translational approaches if they are tackled in a multidisciplinary discourse.

## Ethical considerations and human rights

A paramount challenge in human embryonic development research is navigating the ethical landscape. The use of human preimplantation stage embryos for research has prompted global-scale ethical discussion, including the moral status of the embryo and the rights it should be afforded. Human stem-cell-derived embryo-like structures (embryoids) can nowadays develop a remarkable maturity (Bian et al., 2018; Trujillo et al., 2019). When generating complex neural structures such as mini-brains that sometimes display electrical patterns resembling those of prematurely born babies, we will have to rethink the emergence of mental phenomena such as consciousness.

When we strive to understand more and more the immensely complex interactions between the components of living tissues, one of the greatest challenges will be to closely observe (and to define) the transition between inanimate matter and living beings. This aspect impressively reflects the genuine and close associations between our curiosity regarding our personal origin (i.e., embryonic development) and the most profound philosophical questions about life (Gómez-Márquez, 2023).

Balancing the potential benefits of embryonic research in treating diseases against ethical concerns is a delicate endeavor, necessitating a consensus that respects diverse viewpoints and upholds human dignity. Varying legal frameworks across countries create a fragmented landscape for embryonic development research. Some nations allow extensive research under strict guidelines, while others impose severe restrictions or outright bans. Establishing international standards and regulations that accommodate ethical considerations while fostering scientific advancement is crucial. Such frameworks must also address issues of genome editing technologies, like CRISPR, ensuring responsible use in embryonic research.

## The 3R’s in animal developmental research

Thinking outside the box of human development, we have been able to learn by looking at other organisms: The animal kingdom offers a wealth of stunning mechanisms during development and regeneration that can certainly broaden our mind in general, as well as towards innovative therapeutic approaches. However, our society is searching intensely for new ways for the replacement, reduction and refinement (3R’s) of animal experiments and encourages novel approaches to achieve this goal. While feasible and desirable to a certain extent, especially when considering animal experiments as a surrogate for experiments on human cells, tissues and organs, we should not forget that important mechanisms have been highly conserved during evolution and a considerable part of our knowledge about the molecular mechanisms and control of development in humans results from various well-established model organisms. By stem cell-based novel 3D-approaches like organoids and Organ-on-Chip we cannot only contribute to the reduction of animal experimentation, but at the same time obtain a much more accurate insight into human development that enables disease modelling including personalized medicine. Hence, both the human and non-human approach are contributing in their own way decisively to the elucidation of embryonic development.

## Scientific challenges and technological limitations

Despite major advances, significant scientific challenges remain in fully understanding embryonic development. These include elucidating the complex signaling pathways, genetic factors, and environmental influences that dictate embryonic growth and differentiation. Overcoming technological limitations in imaging, gene editing, and *in vitro* culture systems is vital for advancing our understanding as well as for appropriately addressing developmental disorders and infertility. Last, but not least, human embryonic development is not taught in depth in most regular curricula of medical education and is only sporadically offered to students of biology and related biomedical programs. This inevitably restricts the view of future generations to the three dimensions of adult - often male - human biology and medicine by neglecting the critical time dimension of human life from fertilization to adulthood, quite in contrast to the well-established field of degenerative disease and aging (Burggren and Mueller, 2015).

## Societal implications and interdisciplinary collaboration

Embryonic development research often sparks intense public debate, influenced by cultural, religious, and personal beliefs. Misinformation and misconceptions about the nature and goals of such research can hinder its acceptance and support. Engaging with the public through transparent communication, education, and dialogue is essential to build trust and consensus on the ethical pursuit of embryonic development research.

One of the most eminent challenges related to in depth embryonic development research is its interdisciplinary nature. It is inevitable to crack the discipline boundaries between quite diverse fields of biology, medicine, ethics, and law. Fostering collaboration across these fields is essential for holistic understanding and responsible advancement. Creating platforms for a genuine problem-centered dialogue and cooperation among scientists, ethicists, legal experts, and policymakers will be necessary to facilitate efficient and balanced approaches to research and its applications.

The environmental impact of research practices, including the use and disposal of biological materials, chemicals, climate and resources, needs consideration. Adopting sustainable practices in research laboratories and considering the long-term ecological impacts of scientific advancements in embryonic development are challenges that require innovative solutions and responsible stewardship.

The rapid pace of technological advancement presents both opportunities and challenges. Innovations such as artificial placentas and enhanced genetic editing tools promise to revolutionize our understanding and manipulation of embryonic development. Single-cell sequencing and big data handling require new avenues of qualification for researchers which should not supersede the traditional basic competencies, such as the interpretation of morphological findings. Only a combination of expert views from different angles will enable the scientific community to tackle unforeseen ethical, legal, and social challenges that our societies must be prepared to address in a responsible way.

## New insights into historical disasters affecting human embryonic development

Disruption of embryonic development by exposure to physical and chemical, biological and pharmacological and other noxa the effects of which may currently still be unknown require interdisciplinary efforts for elucidation. The gained knowledge regarding the molecular and cellular mechanisms of teratogenesis will exert a meaningful impact on our society and protect future generations (Vargesson and Hootnick, 2017; Holmes et al., 2018; Vanderhaeghen et al., 2021).

The underlying causes of human malformations can either be genetic alterations or disturbances in gene regulation. The prodigious progress of epigenetics has opened another essential molecular dimension to the elucidation of physiological and teratogenic processes (Walker and Burggren, 2020; Wan et al., 2021) that impressively underscores the importance of a transdisciplinary approach on embryonic development. The latter also comprises genes controlling cell metabolism, survival and proliferation. A key element in the influence of environmental factors that can disturb developmental processes is the oxygen level and resulting production of reactive oxygen species (ROS) in the tissue which in turn can be influenced by pharmacological interventions (Danielsson et al., 2023; Burggren, 2021; Fowden and Forhead, 2015). Catastrophic scenarios such as the teratogenic effect of thalidomide (Vargesson, 2003; 2015; 2023) and Misoprostol/Duogynon (Danielsson et al., 2023), have until now only partially been elucidated, despite them happening several decades ago. Recent challenges in the molecular analysis of developmental defects involve epigenetic mechanisms that unfold their effects even after the exposure has ceased, as well as vertical transmission across generations (Hougaard, 2021). The availability of big data screens and the power of bioinformatical tools hold enormous promise for elucidating many of the open questions soon.

## Implications for balancing disciplines in higher education

Responsibility for our society comprises efforts to train specialists whose competencies contribute to effective interdisciplinary approaches, especially in the biomedical disciplines. This may involve transnational approaches and international networking exceeding the average research collaborations. Young scientists embarking on a career in the field of the molecular control of embryonic development are entering an exciting and dynamic area of research that has far-reaching implications for both scientific understanding and clinical practice. Career prospects for young scientists in this field often start in our medical faculties which have a crucial role in training the next-generation of healthcare professionals and researchers. They must provide comprehensive and up-to-date curricula that incorporate the latest research findings and methodologies on the solid ground of a profound understanding of biological, chemical and physical processes. Only a solid scientific basis can lead to a competent interpretation of the cellular processes in normal and abnormal development (Burggren, 2020). Research departments can foster interdisciplinary connections and provide platforms for knowledge exchange.

Medical faculties could contribute efficiently with great impact by counteracting the growing tendency to eliminate embryology from their curricula and instead actively engage in research related to harmful conditions such as oxidative stress and other threats to physiological embryonic development. This includes conducting clinical trials, translational research, and epidemiological studies to address pressing societal health issues under rapidly changing social and environmental conditions.

Raising awareness of the importance of research on oxidative stress caused by various scenarios and adversely affecting prenatal development is pivotal to combatting its impact on public health. Faculties should educate young scientists also regarding ethical principles and provide guidance on navigating these challenges.

Medical faculties have a vital role to play in nurturing and supporting these scientists, ensuring that their work leads to advancements in maternal and child health and the broader field of medicine. As this field continues to evolve, young scientists will be at the forefront of discoveries that have the potential to transform healthcare and improve the lives of future generations.

## Perspective

The future of embryonic development research holds immense potential for understanding human biology with the goal of preventing or treating a plethora of malformations and serious health conditions. However, navigating the complex web of ethical, legal, scientific, and social challenges requires a multidisciplinary and thoughtful approach. By fostering dialogue, enhancing collaboration, and prioritizing ethical considerations, we can responsibly harness the power of embryonic development research for the betterment of humanity. The journey ahead is fraught with challenges, but with concerted efforts, we can pave the way for a future where scientific advancements and ethical principles coalesce to improve human health and understanding.
